# A GIS Based Approach for Assessing the Association between Air Pollution and Asthma in New York State, USA

**DOI:** 10.3390/ijerph110504845

**Published:** 2014-05-06

**Authors:** Amit K. Gorai, Francis Tuluri, Paul B. Tchounwou

**Affiliations:** 1 Environmental Science & Engineering Group, Birla Institute of Technology, Mesra, Ranchi 835215, Jharkhand, India; 2 Department of Technology, Jackson State University, Jackson, MS 39217, USA; E-Mail: francis.tuluri@jsums.edu; 3 Department of Biology, Jackson State University, Jackson, MS 39217, USA

**Keywords:** air pollution, asthma, health, New York State, GIS

## Abstract

Studies on asthma have shown that air pollution can lead to increased asthma prevalence. The aim of this study is to examine the association between air pollution (fine particulate matter (PM_2.5_), sulfur dioxide (SO_2_) and ozone (O_3_)) and human health (asthma emergency department visit rate (AEVR) and asthma discharge rate (ADR)) among residents of New York, USA during the period 2005 to 2007. Annual rates of asthma were calculated from population estimates for 2005, 2006, and 2007 and number of asthma hospital discharge and emergency department visits. Population data for New York were taken from US Bureau of Census, and asthma data were obtained from New York State Department of Health, National Asthma Survey surveillance report. Data on the concentrations of PM_2.5_, SO_2_ and ground level ozone were obtained from various air quality monitoring stations distributed in different counties. Annual means of these concentrations were compared to annual variations in asthma prevalence by using Pearson correlation coefficient. We found different associations between the annual mean concentration of PM_2.5_, SO_2_ and surface ozone and the annual rates of asthma discharge and asthma emergency visit from 2005 to 2007. A positive correlation coefficient was observed between the annual mean concentration of PM_2.5_, and SO_2_ and the annual rates of asthma discharge and asthma emergency department visit from 2005 to 2007. However, the correlation coefficient between annual mean concentrations of ground ozone and the annual rates of asthma discharge and asthma emergency visit was found to be negative from 2005 to 2007. Our study suggests that the association between elevated concentrations of PM_2.5_ and SO_2_ and asthma prevalence among residents of New York State in USA is consistent enough to assume concretely a plausible and significant association.

## 1. Introduction

Air pollution is one of the most serious environmental threats to urban populations [1]. It has been reported to cause adverse health impacts on people of all ages. Exposure to common urban air pollutants has been linked to a wide range of adverse health outcomes including respiratory and cardiovascular diseases, asthma exacerbation, reduced lung function and premature death [2,3]. Over the past decade, many epidemiologic studies demonstrated positive associations between air pollution and mortality [4,5,6,7,8]. The evidence on adverse effects of air pollution on public health has led to more stringent standards for levels of outdoor air pollutants in many countries including the USA. Asthma is one of the major health issues for all age groups worldwide. Airborne pollutants may influence the symptoms of asthma patients [9,10]. Asthma is a burden on communities, with significant public health and financial consequences. The number of complaints increases day by day due to an increasing trend of air pollution.

Asthma (International Classification of Disease 9th revision, code 493; ICD9-493) [11] is defined by the World Health Organization (WHO) as one of the chronic respiratory diseases. The disease is characterized by bronchial inflammation, with an exaggerated response of the lower airways and limited air flow in these airways. The prevalence of asthma in different countries varies widely, but the disparity is narrowing due to rising prevalence in low and middle income countries and flat trend in high income countries [12]. An estimated 300 million people worldwide suffer from asthma, with 250,000 annual deaths attributed to the disease [13]. Asthma is not only related to genetic and environmental factors, but also it is believed to be affected by air pollutants like PM, ozone, SO_2_. A substantial number of epidemiological studies reported associations between mortality/morbidity with air pollution levels [4,5,6,7,8,14,15,16,17,18,19,20,21,22,23,24,25,26,27]. Inconsistent results have also been reported regarding the association between emergency room visits of acute asthma and air pollution. Though most of the studies reported that air pollution triggers the asthma rate [28,29,30,31]; few reported that there is no correlation between air pollution and asthma rate [25,26,27,32]. 

Asthma is a chronic disease linked with considerable morbidity, mortality, and health care use. The available literature on asthma studies shows a large geographic variation from local/community level all the way to country level. The studies on asthma and other epidemics have raised some important questions as to what factors contribute to the emergence of asthma outbreaks, so there is a dire need to identify those factors and establish the relationship to model the future situations so that problems of public health are minimized if not eradicated. The present study attempts to study the association of air pollution (PM_2.5_, SO_2_, and O_3_) and asthma rate (asthma emergency department visit rate and asthma discharge rate) in New York State during three consecutive years from 2005 to 2007 using GIS. The application of GIS will assist toward better understanding of the problem and its potential solution [33,34]. 

## 2. Study Area

**Figure 1:**
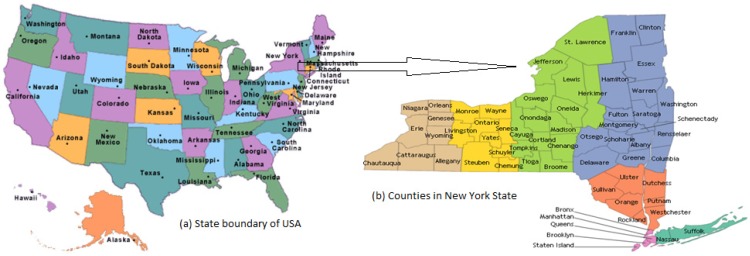
Study area map; (**a**) State boundary of USA; (**b**) Counties in New York State.

In the present work, New York State is selected as the area of study for the analysis and estimation. The study area is shown in Figure 1.

New York is a state in the Northeastern region of the United States. The longitude and latitude of the state are 71°47'25"W to 79°45'54"W and 40°29'40"N to 45°0'42"N respectively. It is the third most populous (19,378,102), and the seventh most densely populated (415.3 inhabitants per square mile) state of the 50 United States [35]. New York covers 54,556 square miles and ranks as the 27th largest state by size [36]. In general, New York has a humid continental climate. Weather in New York is heavily influenced by two continental air masses: a warm, humid one from the southwest and a cold, dry one from the northwest. The winters are long and cold in the Plateau Divisions of the state. In the majority of winter seasons, a temperature of −25 °C or lower can be expected in the northern highlands (Northern Plateau) and 15 °C or colder in the southwestern and east-central highlands (Southern Plateau) [37]. The summer climate is cool in the Adirondacks, Catskills and higher elevations of the Southern Plateau.

## 3. Materials and Methods

Geographic Information Systems (GIS) is an essential scientific tool for health data processing, analysis of geographical distribution and variation of diseases, mapping, monitoring and management of health epidemics [38]. In the past, GIS has been applied to estimate the spatial concentrations of air pollutants [39] and many epidemiologic studies have adopted GIS to explore the health impact of air pollutants on asthma [40,41,42,43]. 

Although different counties in New York State might have varying air pollution concentrations, the air pollution data in each county was not available. In order to make an exposure assessment for the whole of New York State, we linked the annual exposure levels by geostatistical method and corresponding asthma visits to estimate the impact on asthma emergency department visits rate and asthma discharge rate. In the present study, GIS is used to estimate the association between air pollution (fine particulate matter (PM_2.5_), sulfur dioxide (SO_2_) and ozone (O_3_)) and human health (Asthma emergency department visit rate (AEVR) and asthma discharge rate (ADR)). The methodology is applied in two stages to deduce the association between air pollution and asthma rate in New York State. First, we estimated the pollutant level by constructing a spatial model representing a geographical area using daily average pollutant concentration data. Second, we linked air pollutant concentration to asthma rate within the defined study area. All the three pollutants are among the most studied of environmental hazards and are at significant levels in air to adversely affect human health.

### 3.1. Hospital Admission Data

Asthma hospital discharge and asthma emergency department visit data for the period 2005 through 2007 were obtained from Department of Health, New York State’s Asthma Surveillance Summary Report, 2009 [44]. The International Classification of Disease, Ninth Revision, Clinical Modification (ICD-9-CM) diagnosis code 493 [11] was used to identify asthma hospitalization discharge diagnosis. 

New York State, with very high population density, had approximately 19.3 million residents during 2005–2007 [44]. Asthma hospital discharge rate (ADR) indicates the number of asthma-related hospital discharges per 10,000 populations for a specified period of time. Asthma rates for 2005, 2006, and 2007 were calculated by dividing the number of asthma hospital discharges by the estimated population for that time period in a particular zone and then multiplying by 10,000. Similarly, the number of asthma emergency department visit rate (AEVR) indicates the total number of emergency department visits per 10,000 populations for a specified period of time. Both the estimated rates represent crude rate on the basis of estimated population of the county. The county wise population estimates for the year 2005, 2006 and 2007 were obtained from the United States’ Census Bureau [45]. 

**Figure 2:**
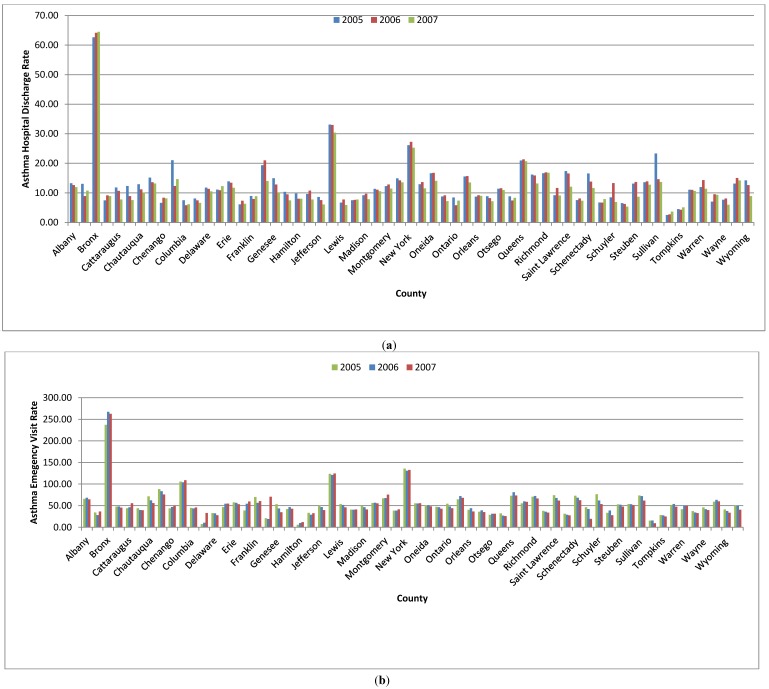
(**a**) County-wise asthma discharge rate in counties of New York State during 2005 to 2007; (**b**) County-wise asthma emergency department visit rate in counties of New York State during 2005 to 2007.

Figure 2a presents the New York State county-specific asthma hospital discharge rates for 2005–2007. Asthma hospital discharge rates vary depending on the region and county of residence. The annual numbers of asthma hospital discharges in New York State were 39,927, 40,205 and 37,950 in 2005, 2006 and 2007, respectively. During this period, the Bronx had the highest asthma discharge rate (62.64 in 2005, 64.16 in 2006, and 64.48 in 2007 per 10,000 residents), followed by Kings County (33.11 in 2005, 32.98 in 2006, and 30.37 in 2007 per 10,000 residents. Similarly, the minimum asthma hospital discharge rate was observed in the county of Tioga (2.52 in 2005, 2.72 in 2006, and 3.68 in 2007 per 10,000 residents).

The annual number of asthma emergency visits in New York State was 159,572, 164,116 and 161,200 in 2005, 2006 and 2007 respectively. Asthma emergency department visit rate of various counties of New York State is represented in Figure 2b for 2005-2007. Spatial trend of asthma emergency department visit rate was found to be different from hospital discharge rate. 

The maximum asthma department visit rate was found in the Bronx county (237.17 in 2005, 267.27 in 2006, and 262.73 in 2007 per 10,000 residents), followed by New York County (135.63 in 2005, 130.72 in 2006, and 132.69 in 2007 per 10,000 residents) in all the three years from 2005 to 2007. The minimum asthma department visit rates were observed as 5.89 per 10,000 population in 2005 in Hamilton County 10.03 in 2006 in the same county and 9.5 in 2007 in Tioga County. 

### 3.2. Air Pollution Data

**Figure 3:**
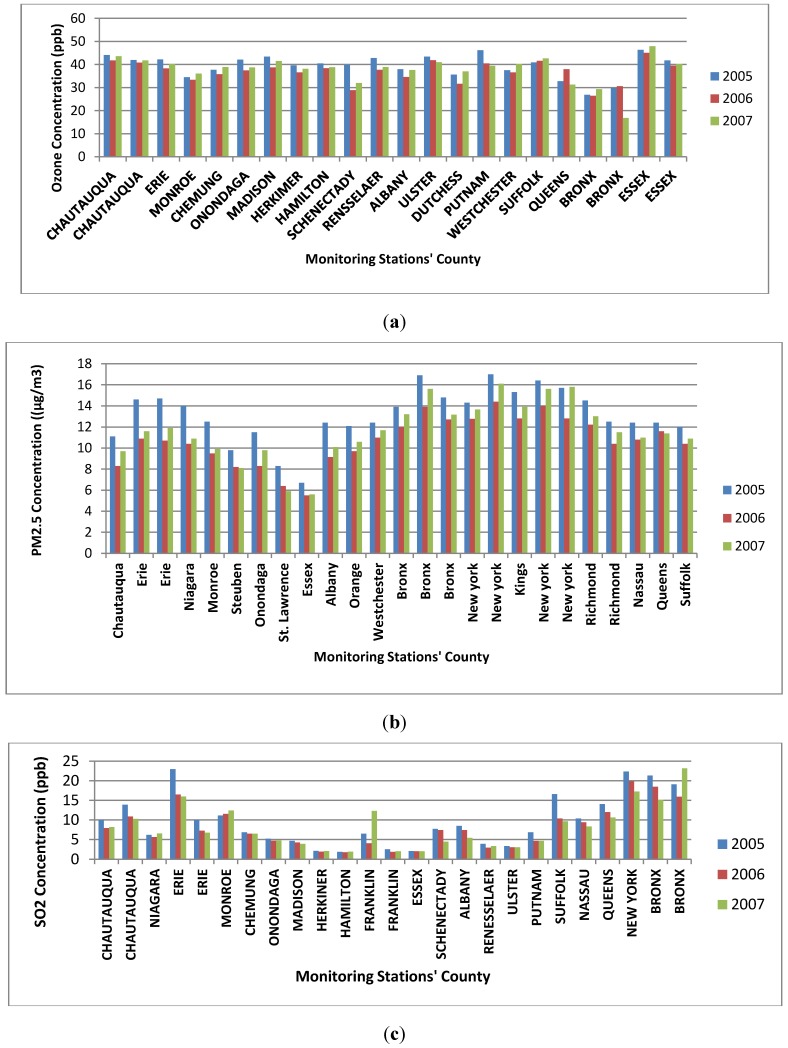
(**a**) Annual average concentration of ozone in various monitoring stations; (**b**) Annual average concentration of PM_2.5_ in various monitoring stations; (**c**) Annual average concentration of SO_2_ in various monitoring station.

Air quality data collected by US EPA’s Air Quality System (AQS) at the various monitoring stations located in different counties of New York State for the three years from 2005 to 2007 were used for the study. The air pollution data used in this study was taken from the United States Environmental Protection Agency (US EPA) air quality system data mart [46]. The pollution concentrations of three criteria air pollutant parameters (SO_2_, PM_2.5_, and O_3_) in various monitoring stations located in different counties were retrieved for a three-year period from 2005 to 2007. Air pollution concentrations of SO_2_, PM_2.5_ and ozone were collected from twenty two, twenty five and twenty five monitoring stations, respectively. The characteristics of the raw data collected from the website are daily average (24 hrs.) concentrations of PM_2.5_, daily maximum 8 hours average concentrations of ozone and daily maximum 1 hour average concentrations of SO_2_. The daily data for each monitoring station were used for determination of annual average concentrations. The annual average concentrations are graphically represented in Figure 3a to Figure 3c respectively for ozone, PM_2.5_ and SO_2_.

The descriptive statistics of the pollutant concentration variations is represented in Table 1. The minimum annual average concentrations of PM_2.5_ for 2005, 2006 and 2007 were 6.7 μg/m^3^, 5.5 μg/m^3^ and 5.6 μg/m^3^ respectively and these values were observed in Essex County. The maximum average concentrations of PM_2.5_ for 2005, 2006 and 2007 were 17 μg/m^3^, 14.4 μg/m^3^ and 16.1 μg/m^3^ respectively and these values were observed in New York City County. 

The maximum values of annual average of maximum 8 hours daily average concentration of O_3_ in 2005, 2006, and 2007 were found to be 46.38 ppb, 45.03 ppb, and 47.93 ppb respectively and these values were observed at the same monitoring station located in Essex County. The minimum values of annual average of maximum 8 hours daily average concentrations of O_3_ in 2005, 2006, and 2007 were found to be 26.84 ppb, 26.41 ppb, and 16.78 ppb respectively and these values were observed in the same county (Essex) but at different monitoring stations.

The maximum values of annual average of maximum 1 hour daily average concentration of SO_2_ in 2005, 2006, and 2007 were found to be 22.99 ppb, 19.86 ppb, and 23.16 ppb respectively and these values were observed at the different monitoring station located in different counties (Erie in 2005, New York in 2006 and Bronx in 2007). The minimum values of annual average of maximum 1 h daily average concentrations of SO_2_ in 2005, 2006, and 2007 was found to be 1.9 ppb, 1.79 ppb, and 1.93 ppb respectively and these values were observed in the same county (Essex) but in different monitoring stations.

**Table 1:** Descriptive statistics of annual average pollution concentrations in New York State.

Items	Minimum	Maximum	Mean	Standard Deviation
**2005**				
Asthma Rate	37.77	94.05	53.04	8.39
Ozone	29.56	44.48	40.21	1.17
PM_2.5_	7.15	16.15	11.39	1.15
SO_2_	2.58	18.65	8.46	2.88
**2006**				
Asthma Rate	37.34	98.30	52.46	9.77
Ozone	30.49	41.92	37.42	1.21
PM_2.5_	5.68	13.30	8.73	1.05
SO_2_	2.14	18.69	6.91	2.31
**2007**				
Asthma Rate	36.56	94.87	50.96	10.43
Ozone	31.75	42.69	39.08	1.76
PM_2.5_	5.73	15.91	9.49	1.08
SO_2_	2.81	13.79	7.18	2.38

## 4. Results and Discussion

### 4.1. Spatial Analysis Using GIS

GIS is an appropriate tool to deduce spatial relationships and to facilitate the proper understanding and resolving of related complex issues. GIS can be used to characterize the sources of pollution and analyze its impact on human health as revealed by numbers of asthma reported to hospital or caused by elevated air pollution. By analyzing the spatial pattern of asthma hospitalization and air pollutants, major threat areas can be visualized in the form of maps with the help of GIS. The use of GIS techniques for statistical analysis and modelling (e.g., pollutants, and diseases) is rarely done in previous studies. However, the past studies have not been fully explored and applied in analyzing the association between exposure of air pollution and human health. During last decade, GIS-based pollution mapping using interpolation techniques such as inverse distance weighting, Kriging and land use regression modeling [33] was explored by many researchers for epidemiological studies. The outbreak of asthma has drawn much attention in the past two decades since data all around the world have shown a high rate of asthma morbidity and mortality despite the availability of effective symptomatic treatment. 

**Figure 4:**
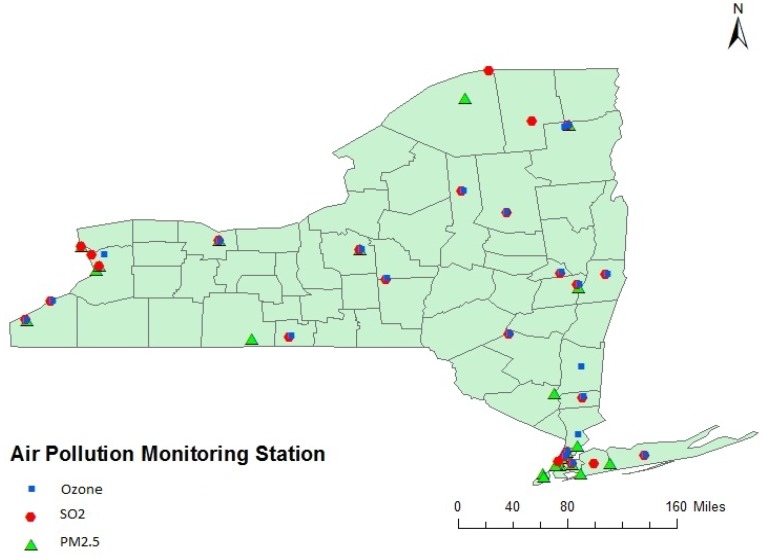
Air Pollution Monitoring Station in New York State.

Measurements of air pollutants were based on data routinely collected at 72 (twenty two for SO_2_, twenty five for PM_2.5_ and ozone) USEPA administered monitoring stations distributed in different counties as represented in Figure 4. All point data were entered into a Geographic Information System using Arcview software from Environmental Systems Research Inc. (ESRI). The first stage involved determining the location (latitude and longitude) of air pollution monitoring stations. The spatial location (latitude and longitude) of air pollution monitoring stations were also obtained from the same source of air pollution monitoring website. The concentrations of SO_2_, O_3_, and PM_2.5_ are reported as daily maximum 1 hour average concentrations, daily maximum 8 h average concentrations and daily 24 h average concentrations respectively. Annual average concentrations were calculated using the daily average value in a particular year for each of the monitoring station. The spatial locations of each of the selected monitoring stations along with the pollutant concentrations were fed into the GIS system. With the point data from each monitoring station, the Ordinary Kriging (OK) method was used to estimate the spatial distribution of pollutant levels in each county from 2005 to 2007 for each of the three pollutants: SO_2_, O_3_, and PM_2.5_. Analyses were carried out by Ordinary Kriging method using the Geostatistical Analyst Extension of ArcGIS. In Kriging, a smooth surface is estimated from irregularly spaced data points based on the assumptions that the spatial variation in the feature (O_3_, PM_2.5_, and SO_2_) is homogeneous over the domain depends only on the distance between sites. 

In general, the Kriging method [39,47] was used as a statistical mapping technique for using data collected at each point location, to predict concentration in each grid cell over a spatial domain. This paper used stable kind of semivariogram model for prediction of pollution concentration. Mean standardized error (MSE) and root mean square standardized (RMSSE) were used to select accuracy of the model fit which estimate the distribution of air pollutants. Partial sill, range and nugget values of the selected error were determined to represent the semivariogram model characteristics. The parameters of semivariogram model along with the errors outcome of each individual model are listed in Table 2. The cross-validation of the four air pollutants was done manually by ArcGIS Geostatistical extension. The criteria for a good fitting Kriging model used in this study were an average MSE near 0 and RMSS near 1. According to the cross validation results, the variance of PM_2.5_, SO_2_ and O_3_ was little bit overestimated as the RMSS value was less than 1 [48]. Since, we are more emphasized on the relative concentration level and its association with asthma rate; it doesn’t influence much on the final outcome.

**Table 2:** Parameters of semivariogram model.

Parameter	Fitted Model	Nugget	Partial Sill	Lag Size (in degree)	Range(in degree)	MSE	RMSSE
**Ozone**							
2005	Stable	0	29.78	0.32	3.79	−0.03	0.80
2006	Stable	0	30.11	0.08	0.66	−0.006	0.83
2007	Stable	9.66	16.72	0.08	0.67	0.001	0.77
**PM_2.5_**							
2005	Stable	0.85	6.52	0.30	2.46	0.09	0.76
2006	Stable	0.72	7.68	0.34	2.70	0.07	0.68
2007	Stable	0.01	8.13	0.30	2.41	0.08	0.69
**SO_2_**							
2005	Stable	0	31.79	0.17	1.38	0.00	0.83
2006	Stable	0	24.14	0.17	1.42	0.02	0.77
2007	Stable	21.49	13.22	0.26	2.14	−0.01	0.85

**Figure 5:**
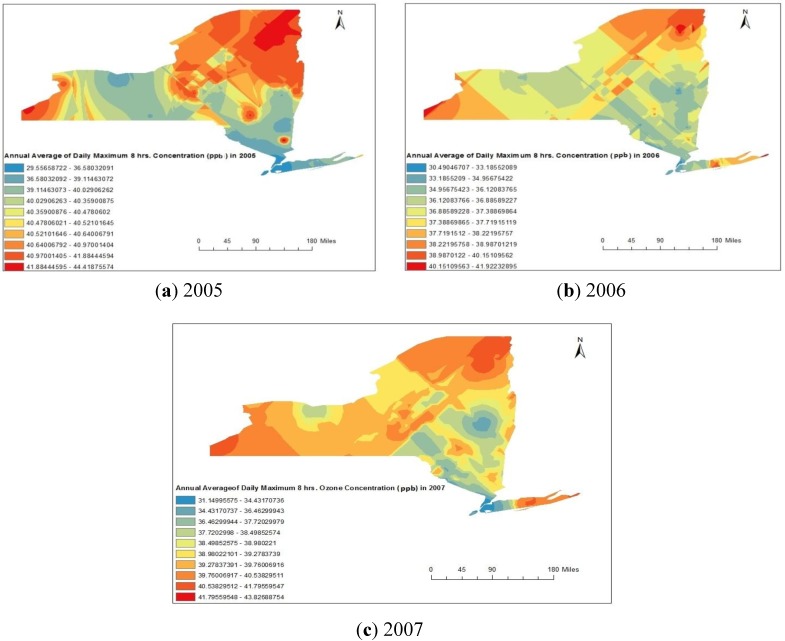
(**a**–**c**) Spatial distribution of annual average maximum daily eight hours ozone concentrations during 2005 to 2007.

In this study, ambient O_3_, PM_2.5_, and SO_2_ levels for each county within the New York State were estimated because these values could be linked to the residences of asthma rate obtained from recorded and interpolated data. The spatial distribution maps for the O_3_ obtained from Kriging method are represented in Figure 5a–c respectively for 2005, 2006 and 2007. Figure 5a–c clearly indicate that the concentrations were maximum in the counties situated in north-east part (Clinton, Franklin, St. Lawrence and Essex) and southern part counties (Chautauqua) of the New York State, USA. The minimum concentrations were observed in some parts of the central region (Monroe) and south-east corner counties (Bronx, and Queens) of the New York State, USA.

**Figure 6:**
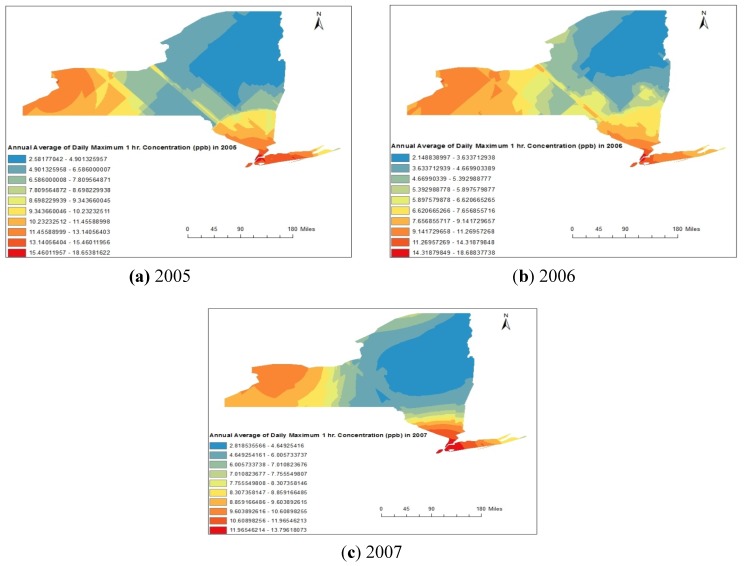
(**a**–**c**) Spatial distribution of annual average maximum daily one hour SO_2_ concentrations during 2005 to 2007.

Similarly, the spatial distribution maps for SO_2_ obtained from Kriging method are represented in Figure 6a–c respectively for 2005, 2006 and 2007. 

Spatial distribution of annual average concentration represented in Figure 2a–c clearly indicates that the concentration values were maximal in the counties situated in western part (Erie, Niagara, and Chautauqua) and south-eastern part counties (Bronx, and Queens) of New York State, USA. The minimum concentrations were observed in north-eastern part (Clinton, Franklin, Hamilton and Essex) and extended to central region of New York State, USA.

**Figure 7:**
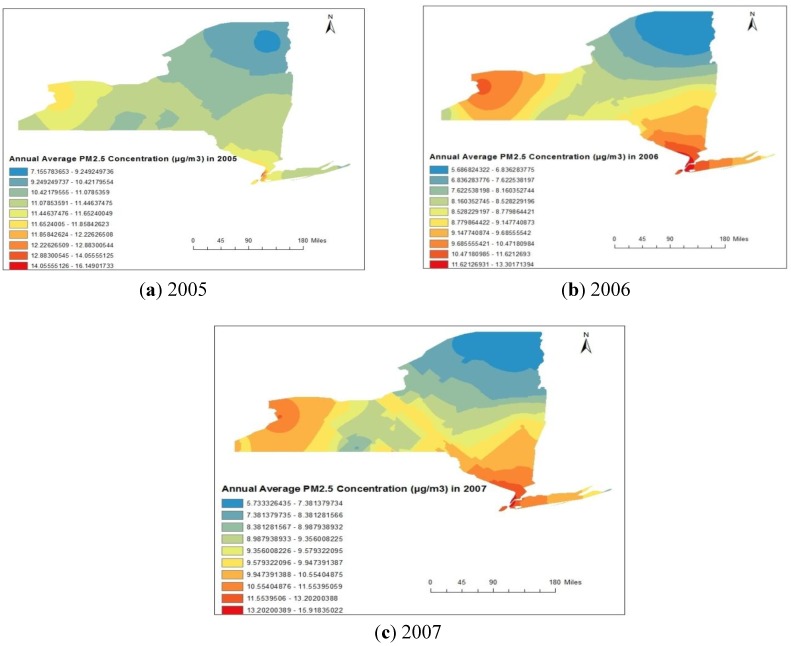
(**a**–**c**) Spatial distribution of annual average 24 h PM_2.5_ concentrations during 2005 to 2007.

The spatial distribution maps for PM_2.5_ obtained from Kriging method are represented in Figure 7a–c, respectively, for 2005, 2006 and 2007. Spatial distribution of annual average concentrations represented in Figure 7a–c indicates more or less similar distribution as that of the SO_2_ distribution but reverse spatial trend from the O_3_ distribution. That is the concentration values were maximum in the counties situated in western part (Erie) and south-eastern part (Bronx, and Queens) of New York State, USA. The minimum concentrations were observed in north-eastern part (Clinton, Essex and Franklin) of New York State, USA. The spatial distributions of each of the individual pollutants were more or less same in each of the three years from 2005 to 2007.

**Figure 8:**
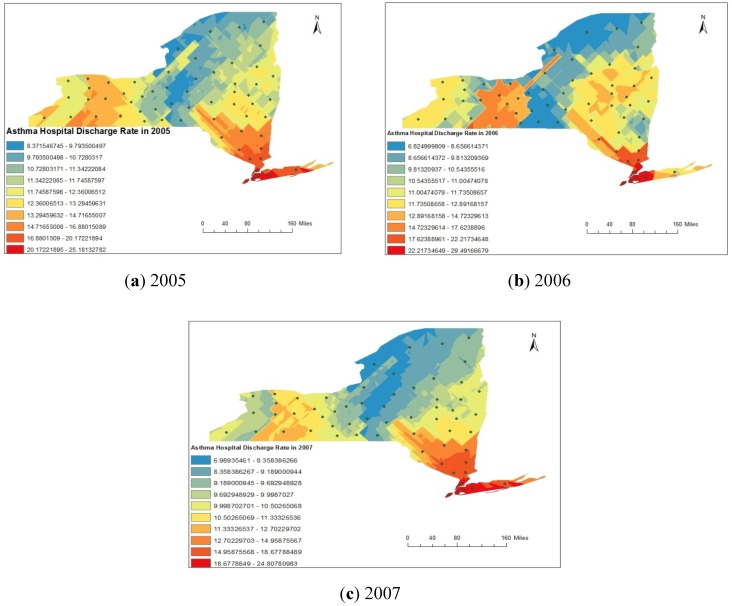
(**a**–**c**) Spatial distribution of asthma discharge rate (ADR) during 2005 to 2007.

**Figure 9:**
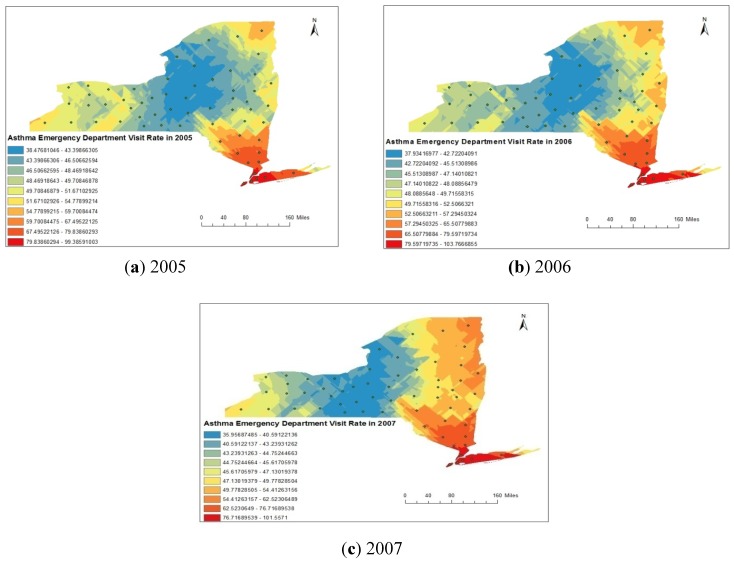
(**a**–**c**). Spatial distribution of asthma emergency department visit rate (AEVR) during 2005 to 2007.

For the distribution analysis of asthma rate (asthma discharge rate and emergency department visit rate), we have designed a points shape file by considering a location at the centroid of each county of New York State. The attributes entered to particular centroid point were the asthma rate calculated for same county. Subsequently, the raster images for asthma discharge rate and emergency department visit rate were created by interpolation using the Kriging method. The spatial distributions of asthma discharge rate obtained from Kriging analysis are represented in Figure 8a–c for the year 2005, 2006 and 2007 respectively. Spatial distribution of asthma discharge rate represented in Figure 8a–c clearly indicates that rate was maximum in south-eastern part of the state and minimum western part of the state. Similar spatial trend was observed in each of the three years from 2005 to 2007. Similarly, the spatial distributions of asthma emergency visit department rate obtained from Kriging analysis are represented in Figure 9a–c, respectively, for the year 2005, 2006 and 2007. Spatial distribution of asthma discharge rate represented in Figure 9a–c clearly indicates that the maximum rate was found in the south-eastern part of the State and the minimum in the central region.

### 4.2. Statistical Analyses

The study sought to investigate the spatio-temporal association between air pollutants and asthma rate. To understand the inter-relationships among predictor variables, both GIS based correlation analysis (map correlation analysis) and point data correlation analyses were carried out. We conducted Pearson Correlation analysis among the pollutant variables and asthma rates. 

#### 4.2.1. Map Correlation Analysis

Spatial distribution maps of ambient air pollution (SO_2_, O_3_, and PM_2.5_) and asthma rates (AEVR and ADR) were used for correlation analysis in GIS on year wise basis. The map correlation results for three years (2005, 2006, and 2007) are reported in Table 3. Map correlation results clearly indicate that PM_2.5_ and SO_2_ are positively correlated with asthma rate whereas ozone has a negative correlation with asthma rate.

**Table 3:** Map Correlation Statistics.

2005					
	AEVR	ADR	O3	PM_2.5_	SO_2_
AEVR *	1.00				
ADR **	0.84	1.00			
**O_3_**	−0.54	−0.55	1.00		
**PM_2.5_**	0.29	0.39	−0.56	1.00	0.82
**SO_2_**	0.46	0.52	−0.59	0.82	1.00
**2006**					
	**AEVR**	**ADR**	**O_3_**	**PM2.5**	**SO_2_**
**AEVR**	1.00				
**ADR**	0.54	1.00			
**O_3_**	−0.25	−0.25	1.00		
**PM_2.5_**	0.39	0.46	−0.41	1.00	
**SO_2_**	0.31	0.38	−0.30	0.86	1.00
**2007**					
	**AEVR**	**ADR**	**O_3_**	**PM_2.5_**	**SO_2_**
**AEVR**	1.00				
**ADR**	0.86	1.00			
**O_3_**	−0.42	−0.49	1.00		
**PM_2.5_**	0.25	0.53	−0.48	1.00	
**SO_2_**	0.13	0.41	−0.33	0.73	1.00

Notes: * AEVR: Asthma Emergency Department Visit Rate; ** ADR: Asthma Hospital Discharge Rate.

Results of the map correlation analyses reveal that AEVR and ADR are negatively correlated with the ground level ozone (O_3_) concentration. All the three years showed the same trend. The correlation values of ozone concentration and the AEVR are −0.54, −0.25 and −0.25 in 2005, 2006 and 2007 respectively. Similarly, the correlation values between ADR and ozone concentration are −0.55, −0.25, and −0.49 in 2005, 2006 and 2007 respectively. But the reverse trend was observed with PM_2.5_ and SO_2_ concentrations in each of the three years from 2005 to 2007. PM_2.5_ is significantly correlated with both the factors AEVR and ADR. The correlation values between PM_2.5_ and AEVR are 0.29, 0.39, and 0.25 in 2005, 2006, and 2007 respectively. Similarly, the correlation values between PM_2.5_ and ADR are 0.39, 0.46, and 0.53 in 2005, 2006, and 2007 respectively. Map layers correlation results also indicate that the spatial distribution of SO_2_ concentrations is positively correlated with both the factor AEVR and ADR. The correlation values between spatial distribution of SO_2_ and ADR are 0.52, 0.38, and 0.41 in 2005, 2006, and 2007 respectively. Similarly, the correlation values between spatial distribution of SO_2_ and AEVR are 0.46, 0.31, and 0.13 in 2005, 2006, and 2007 respectively. 

#### 4.2.2. Point Correlation Analysis

The asthma rates (asthma discharge rate and asthma emergency department visit rate) were compared with the extracted values of pollutant concentrations (SO_2_, PM_2.5_ and O_3_) for understanding the association between asthma and air pollution. Asthma rate at the specified centroid position of each county was considered the same as that of the corresponding county value. Since the monitoring values of pollutant concentrations at the same location were not available, they were extracted from the interpolated map using GIS. The extracted values of pollutant concentrations (O_3_, PM_2.5_, and SO_2_) at the centroid point of each of the county are represented in Table 4. The data represented in Table 4 for three years (2005, 2006, and 2007) were used for correlation analyses to determine the correlation coefficients values. The correlation analyses were carried out using Pearson two tailed correlation analysis using SPSS Statistics software version 21. Correlation analyses results are represented in Table 5. 

The results represented in Table 5 clearly indicate that the associations or relations of the asthma rates (ADR and AEVR) with pollutant concentrations (PM_2.5_, O_3_, and SO_2_) are similar to those obtained from map correlation analyses. That is ozone concentration showed a negative correlation with both asthma discharge rate and asthma emergency visit rate while the other two pollutants (SO_2_ and PM_2.5_) showed a positive correlation. 

The correlation coefficients for ozone concentration and the AEVR are −0.638, −0.479 and −0.400 for 2005, 2006 and 2007 respectively. These correlation coefficients are significant at 1% level. Similarly, the correlation coefficients between ADR and ozone concentration are −0.575, −0.490, and −0.455 for 2005, 2006 and 2007 respectively. But the reverse trend was observed with PM_2.5_ and SO_2_ concentration in each of the three years from 2005 to 2007. PM_2.5_ is significantly correlated with both AEVR and ADR. The correlation coefficients between PM_2.5_ and AEVR are 0.395, 0.511, and 0.343 for 2005, 2006, and 2007 respectively. Similarly, the correlation coefficients for PM_2.5_ and ADR are 0.371, 0.505, and 0.431 in 2005, 2006, and 2007 respectively. Correlation data also indicate that the SO_2_ concentration is positively correlated with both the factor AEVR and ADR. The correlation coefficients for SO_2_ and ADR are 0.449, 0.470, and 0.509 in 2005, 2006, and 2007 respectively. Similarly, the correlation coefficients between SO_2_ and AEVR are 0.448, 0.446, and 0.469 in 2005, 2006, and 2007 respectively. Year-wise variation in correlation coefficients between asthma rate (ADR and AEVR) and annual average pollutant concentration (PM_2.5_, SO_2_, and Ozone) indicates a consistent trend.

According to the past research, the majority of researchers considered air pollutants a risk factor for asthma, although the roles of specific air pollutants on various respiratory illnesses remain unclear [49,50]. That is, the general effect of air pollution leads to adverse respiratory events. But, the effect of specific air pollutants on asthma rate is yet to be examined for a plausible conclusion. According to Chan *et al.* [51] PM_10_ might have a positive impact on asthma outpatient and emergency settings. They further suggested that future research is required to validate robust spatiotemporal patterns and trends. A study conducted by Nawahda [32] suggested that the association between elevated concentrations of PM_2.5_ and surface ozone and asthma prevalence among school children in Japan is not strong enough to assume concretely a plausible and significant association. On the other hand, a controlled laboratory study conducted by Koenig [52] revealed that ozone aggravates asthma. Thus, more specific studies are needed for understanding the role of specific air pollutants on asthma rate. These studies should also consider the socioeconomic factors and climatic factors for better reflections.

**Table 4:** Asthma rate at the centroid of each county along with extracted pollutant concentration from interpolated maps.

County Name	ADR			AEVR			Ozone Concentration (ppb)			PM_2.5_ Concentration (µg/m^3^)			SO_2_ Concentration (ppb)		
	2005	2006	2007	2005	2006	2007	2005	2006	2007	2005	2006	2007	2005	2006	2007
Clinton	21.04	12.36	14.66	105.55	103.94	108.77	42.38	39.91	41.97	8.45	6.04	6.94	3.20	2.40	4.18
Franklin	8.97	7.96	8.88	69.84	56.49	60.45	41.54	38.86	40.49	8.63	6.09	7.04	3.67	2.82	4.88
Essex	6.10	7.34	6.35	38.66	54.19	59.69	42.02	38.08	39.41	8.56	6.21	7.13	3.66	3.05	3.21
Hamilton	9.82	8.02	8.05	5.89	10.03	12.07	41.04	37.67	39.62	9.97	7.24	8.14	4.03	3.05	3.06
Herkimer	9.64	10.78	7.77	33.44	29.05	32.48	40.87	36.37	38.18	10.50	7.75	8.70	4.00	3.40	3.25
Washington	7.04	9.56	9.36	37.14	34.25	32.83	41.15	38.31	40.23	10.46	8.06	8.72	4.16	3.64	3.68
Warren	11.96	14.34	11.41	41.56	50.95	50.05	41.28	37.37	39.31	9.80	7.40	8.07	4.01	3.45	3.14
Saratoga	17.38	16.58	12.10	74.10	67.59	61.48	41.12	36.43	39.01	10.96	8.31	9.13	4.28	4.07	3.57
Fulton	19.35	20.97	10.06	20.98	19.16	70.46	40.67	36.06	38.10	10.76	8.16	8.98	4.50	3.66	3.22
Montgomery	12.32	12.87	11.45	66.86	67.37	75.51	40.53	35.97	38.09	10.99	8.43	9.17	4.83	4.15	3.66
Rensselaer	16.21	15.89	13.21	55.22	59.82	58.71	40.64	36.38	38.81	11.63	8.91	9.66	6.32	4.90	4.67
Schenectady	7.56	8.11	7.37	31.41	29.07	27.53	40.30	35.67	39.10	11.35	8.69	9.41	5.02	4.99	4.03
Otsego	11.42	11.58	10.97	28.70	31.57	31.31	40.13	36.36	35.98	11.00	8.57	9.28	7.37	5.92	4.08
Schoharie	16.58	13.84	11.69	72.84	68.13	62.52	40.61	36.10	38.10	11.25	8.76	9.42	4.89	4.22	4.14
Albany	13.28	12.63	11.95	65.72	68.16	64.60	39.90	35.72	38.23	11.70	8.98	9.69	6.91	6.04	4.39
Delaware	11.78	11.39	10.53	32.87	32.32	27.86	40.14	35.54	34.66	11.19	8.91	9.50	7.58	6.15	5.09
Columbia	7.53	5.83	6.15	44.89	43.20	45.72	39.36	36.12	38.20	11.82	9.20	10.01	7.60	6.24	5.39
Greene	10.38	9.49	7.47	41.92	46.45	42.80	40.22	36.39	38.22	11.65	9.11	9.81	6.82	5.60	5.11
Ulster	11.07	11.05	10.67	51.52	53.76	47.31	39.92	36.78	36.60	11.83	9.40	10.19	9.71	7.09	6.81
Dutchess	11.11	10.89	12.33	46.41	54.32	54.69	37.66	35.17	39.37	11.95	9.47	10.37	10.05	7.50	7.79
Sullivan	23.31	14.63	13.72	73.59	72.12	61.55	39.19	35.33	36.09	11.96	9.58	10.35	10.63	8.21	7.27
Orange	15.55	15.70	13.54	64.58	72.01	68.04	38.90	36.30	35.64	12.42	10.47	10.96	11.23	8.73	9.74
Putnam	8.84	7.45	8.35	32.44	21.17	25.94	41.49	37.18	36.30	12.24	10.01	10.84	10.79	7.09	9.52
Westchester	13.15	15.04	14.23	59.00	63.31	59.89	38.22	36.12	35.29	12.73	10.74	11.50	12.26	9.70	10.97
Rockland	9.21	11.69	9.12	37.69	36.17	34.01	37.53	35.78	35.04	13.29	11.35	12.03	12.23	10.16	11.37
Bronx	62.64	64.16	64.16	237.17	267.27	262.73	31.92	33.21	33.73	13.94	12.20	12.91	16.04	14.65	12.51
Nassau	14.92	14.26	13.57	38.35	38.41	41.19	36.11	36.16	33.35	12.35	10.79	11.18	13.09	10.21	12.11
New York	26.09	27.25	25.27	135.63	130.72	132.69	32.14	32.45	32.40	15.41	13.15	14.55	16.35	16.25	12.88
Queens	20.95	21.32	20.67	72.92	81.13	73.35	33.48	35.39	33.17	13.04	11.81	11.67	15.18	12.78	12.83
Suffolk	13.61	13.91	12.76	53.52	53.06	51.12	39.01	37.46	39.76	11.43	9.64	10.15	11.74	9.00	9.23
Kings	33.11	32.98	30.37	123.53	121.08	124.33	33.82	34.46	33.50	14.42	12.28	13.29	15.28	13.93	13.27
Richmond	16.67	16.98	16.82	70.52	72.36	66.42	35.49	33.62	32.13	13.37	11.37	11.93	14.58	12.68	13.40
St. Lawrence	6.54	6.25	5.36	33.54	38.61	27.91	41.04	38.27	40.22	9.37	6.74	7.22	5.05	4.11	5.90
Jefferson	8.64	7.57	6.08	49.79	46.46	39.54	40.80	37.12	38.92	10.37	7.86	8.35	5.75	5.15	5.56
Lewis	6.72	7.78	5.91	53.41	51.48	45.78	40.84	37.13	38.92	10.25	7.61	8.33	5.24	4.28	4.46
Oswego	8.89	8.25	7.20	36.20	39.07	35.10	40.99	37.14	38.93	11.07	8.25	9.09	5.89	5.08	5.62
Oneida	16.65	16.78	14.07	50.45	51.23	48.23	41.20	38.09	39.52	10.81	8.07	9.02	4.26	3.71	3.99
Cayuga	12.33	8.90	7.57	43.77	39.81	39.32	40.24	36.69	39.09	11.47	8.51	9.37	7.28	6.34	6.72
Niagara	12.96	13.65	11.54	55.12	54.92	55.38	40.49	37.25	39.25	13.55	10.41	10.76	11.75	9.58	9.90
Orleans	8.74	9.21	9.00	40.25	43.76	33.22	40.28	36.99	39.23	12.82	10.02	10.29	11.68	9.67	10.25
Monroe	11.36	11.09	10.55	56.10	56.76	55.12	38.66	36.49	39.08	12.42	9.57	9.95	10.21	9.28	9.68
Wayne	7.68	8.12	5.99	45.88	41.67	39.45	39.43	36.53	39.07	11.66	8.85	9.37	7.71	7.31	8.11
Onondaga	8.79	9.18	7.24	47.06	46.08	42.73	40.97	36.87	39.10	11.55	8.41	9.75	8.37	6.43	6.14
Madison	8.40	9.72	7.84	49.39	46.22	40.99	40.60	37.27	36.38	11.18	8.32	9.39	5.51	4.71	4.84
Genesee	14.98	12.85	10.18	53.27	43.23	35.04	40.26	36.99	39.23	12.81	10.03	10.30	11.68	9.66	9.96
Erie	13.92	13.46	11.75	57.63	56.42	53.08	40.59	38.06	40.10	13.86	10.54	11.13	11.70	9.27	9.80
Ontario	8.44	5.83	7.41	54.29	48.07	43.81	39.34	37.05	39.50	11.64	9.01	9.39	9.97	8.66	8.92
Seneca	8.47	13.33	6.95	76.27	61.86	53.46	39.96	36.61	39.08	11.44	8.60	9.31	7.40	6.59	7.81
Livingston	7.50	7.65	7.79	40.72	40.55	41.09	39.76	36.92	39.12	11.94	9.39	9.58	10.13	8.68	9.48
Wyoming	14.26	12.65	8.94	40.91	37.49	33.87	40.41	37.04	39.23	12.71	9.93	10.26	11.67	9.54	9.99
Cortland	8.11	7.48	6.65	7.50	10.52	33.05	40.67	36.68	38.75	11.35	8.48	9.50	5.57	5.19	5.12
Yates	50.14	49.55	40.42	50.14	49.55	40.42	39.71	37.18	39.51	11.30	8.67	9.15	9.79	8.26	8.21
Chenango	6.65	8.37	8.16	43.59	46.90	49.94	40.63	37.72	39.20	11.65	8.53	9.84	6.90	6.10	4.94
Tompkins	4.53	4.32	5.10	28.26	27.60	24.92	40.14	36.66	38.94	11.31	8.44	9.33	6.61	5.84	6.36
Steuben	13.15	13.71	8.73	52.80	51.99	47.59	40.00	36.99	39.12	10.97	8.57	9.02	10.11	8.16	8.80
Chautauqua	12.93	11.21	9.74	71.54	62.22	55.65	41.34	38.64	40.16	11.94	8.81	10.01	11.48	9.24	9.26
Schuyler	6.76	6.74	7.90	46.11	41.95	19.46	39.74	37.16	39.52	11.06	8.43	9.10	9.12	7.40	7.93
Cattaraugus	11.82	10.70	7.77	43.88	46.35	55.39	40.32	38.05	40.10	12.62	9.66	10.26	11.46	9.30	9.53
Allegany	13.06	8.91	10.80	34.36	28.36	36.68	40.40	37.24	39.29	12.04	9.28	9.84	10.64	8.80	9.23
Broome	7.48	9.16	8.91	47.59	47.98	45.25	40.03	37.29	38.94	11.60	8.67	9.79	5.48	5.20	4.85
Tioga	2.52	2.72	3.68	15.50	15.91	9.50	39.93	37.48	39.18	10.99	8.58	9.07	5.60	5.44	5.48
Chemung	15.19	13.64	13.20	88.23	83.40	75.70	38.97	36.97	39.43	11.46	8.52	9.64	7.08	6.45	6.55

**Table 5:** Correlation matrix of asthma rate and air pollutants.

2005					
	ADR	AEVR	Ozone	SO_2_	PM_2.5_
**ADR**	1				
**AEVR**	749 **	1			
**Ozone**	−0.575 **	−0.638 **	1		
**SO_2_**	0.449 **	0.448 **	−0.759 **	1	
**PM_2.5_**	0.371 **	0.395 **	−0.709 **	0.868 **	1
**2006**					
	**ADR**	**AEVR**	**Ozone**	**SO_2_**	**PM_2.5_**
**ADR**	1				
**AEVR**	0.765 **	1			
**Ozone**	−0.490 **	−0.479 **	1		
**SO_2_**	0.470 **	0.446 **	−0.716 **	1	
**PM_2.5_**	0.505 **	0.511 **	−0.608 **	0.922 **	1
**2007**					
	**ADR**	**AEVR**	**Ozone**	**SO_2_**	**PM_2.5_**
**ADR**	1				
**AEVR**	0.822 **	1			
**Ozone**	−0.455 **	−0.400 **	1		
**SO_2_**	0.509 **	0.469 **	−0.741 **	1	
**PM_2.5_**	0.431 **	0.343 **	−0.535 **	0.794 **	1

Note: ** Correlation is significant at the 0.01 level (2-tailed).

## 5. Limitations of the Study

GIS facilitates the research studies for association between air pollution and health more quickly and with less effort but data quality, lack of spatial detail and spatial consistency between data sets impede their utility. The major limitations of the GIS based studies include the availability of uniformly distributed pollution and health data. A number of data problems and data limitations are encountered with the integration of health data in GIS. Primarily, the interpolation analysis gives better pollution level prediction for uniformly distributed spatial data in the study area. But, the pollution monitoring stations in New York State was not uniformly distributed and this may lead to some errors in prediction level. 

A major drawback to the health data used in this analysis is that asthma hospitalization records only provide number of asthma cases, and do not reflect the severity of the asthma problem. Again, there is no statewide reporting of asthma and therefore no centralized asthma database. People suffering from asthma may be seen by a private doctor or may not be seen by any health care provider. This type of cases may not be listed in the asthma database. Furthermore, asthma data available does not consider the movement of people from one county to the next. This study used annual average level of pollutants at a particular location as the population's exposure level but the workplace was not located in the same location and this could be bias about an individual’s exposure estimation, which could influence the results. In addition, the counties where outpatient visits and emergency visits took place were assumed to be the same districts where people were exposed to pollution. This may not always have been the case. The true exposure time was difficult to estimate due to lack of exposure information.

## 6. Conclusions

We found significant associations between exposure to modeled SO_2_ and PM_2.5_ with asthma prevalence rate in New York State, USA. Asthma prevalence among the residents was closely associated with the exposure of PM_2.5_ followed by SO_2_. But there was a negative association between asthma rate and ground level ozone concentration. In conclusion, this preliminary study illustrates the potential use of the GIS-based method to evaluate the effects of air pollution on asthma rate (asthma emergency visit rate and asthma discharge rate). The results of this study provide a better understanding of the correlation of air pollution with asthma patient visits, and demonstrate that SO_2_, and PM_2.5_ might have a positive impact each on asthma emergency visit rate (AEVR) and asthma discharge rate (ADR) while O_3_ might have a negative impact. Since many other pollutants such as NO_2_, and VOCs may cause adverse health outcomes, future research is required to provide robust spatiotemporal patterns and trends.
